# Retracted: Mutations in KCNQ potassium channels cause pharyngeal pumping defects in *C. elegans*

**DOI:** 10.17912/micropub.biology.000165

**Published:** 2019-08-23

## Description

Also as a matter of record this article was originally published with an error in one of the author’s names, B. Weeks should be B. Roberts. This has been corrected for the citation.

https://www.micropublication.org/journals/biology/w2mw2d/ published online Nov 10, 2016 @ 16:07, retracted Aug 23, 2019.

The authors have identified erroneous data reported in this article. Thus, in order to correct the scientific record the authors have retracted this microPublication in its entirety.

